# Effect of Tree-to-Shrub Type Conversion in Lower Montane Forests of the Sierra Nevada (USA) on Streamflow

**DOI:** 10.1371/journal.pone.0161805

**Published:** 2016-08-30

**Authors:** Ryan R. Bart, Christina L. Tague, Max A. Moritz

**Affiliations:** 1 Earth Research Institute, University of California Santa Barbara, Santa Barbara, California, United States of America; 2 Bren School of Environmental Science and Management, University of California Santa Barbara, Santa Barbara, California, United States of America; 3 Department of Environmental Science, Policy, and Management, University of California, Berkeley, California, United States of America; Oregon State University, UNITED STATES

## Abstract

Higher global temperatures and increased levels of disturbance are contributing to greater tree mortality in many forest ecosystems. These same drivers can also limit forest regeneration, leading to vegetation type conversion. For the Sierra Nevada of California, little is known about how type conversion may affect streamflow, a critical source of water supply for urban, agriculture and environmental purposes. In this paper, we examined the effects of tree-to-shrub type conversion, in combination with climate change, on streamflow in two lower montane forest watersheds in the Sierra Nevada. A spatially distributed ecohydrologic model was used to simulate changes in streamflow, evaporation, and transpiration following type conversion, with an explicit focus on the role of vegetation size and aspect. Model results indicated that streamflow may show negligible change or small decreases following type conversion when the difference between tree and shrub leaf areas is small, partly due to the higher stomatal conductivity and the deep rooting depth of shrubs. In contrast, streamflow may increase when post-conversion shrubs have a small leaf area relative to trees. Model estimates also suggested that vegetation change could have a greater impact on streamflow magnitude than the direct hydrologic impacts of increased temperatures. Temperature increases, however, may have a greater impact on streamflow timing. Tree-to-shrub type conversion increased streamflow only marginally during dry years (annual precipitation < 800 mm), with most streamflow change observed during wetter years. These modeling results underscore the importance of accounting for changes in vegetation communities to accurately characterize future hydrologic regimes for the Sierra Nevada.

## Introduction

Forest ecosystems in the western U.S. and throughout the world are in a state of transition [1,2]. Rising temperatures associated with climate change are increasing atmospheric water demands on vegetation [3], increasing the severity of droughts [4], and altering the timing of water availability through decreases in mountain snowpack [5,6]. At the same time, wildfires in many areas are becoming more frequent and more widespread [7,8], while bark beetles are exploiting warmer temperatures to increase rates of tree invasion [9]. These processes are testing the resilience of many forest ecosystems, and higher rates of tree mortality are being observed in many western U.S. forests [10–12].

In lower montane forests of California, there is increasing evidence that some forests are not regenerating, particularly following stand-replacing wildfires [13,14]. Instead, the dominant species on these landscapes are converting from mixed conifers to sclerophyll shrublands [14–18]. Shrublands are more resilient to water stress than forests and in some cases, may become permanently established due to pyrogenic feedbacks associated with higher fire frequencies [19,20]. It is also likely that shrub conversion may be more prevalent on equatorial-facing aspects due to higher fire frequencies and severities [21].

Tree-to-shrub type conversion in lower montane forest may alter vegetation properties such as leaf area index, rooting depth, and stomatal conductance; which in turn may affect numerous ecohydrologic processes including vegetation interception and evaporation, vegetation transpiration, and streamflow [22]. As mountainous regions in California are key sources of water supply for urban, agriculture and environmental purposes [23], understanding type conversion effects on the these processes, and in particular streamflow, is critical.

Climate change also impacts Sierra Nevada streamflow and the direct hydrologic effects of climate warming are well documented. As temperatures increase with climate change, a shift in precipitation regime from snow to rain impacts the cycling of water through a watershed, altering processes such as snowmelt, forest transpiration, and streamflow [6,24–26]. For the latter process, numerous studies have predicted earlier shifts in Sierra Nevada streamflow timing [27–29] and reductions in spring/summer hydropower production [30,31]. However, while these and other studies have investigated the effect of climate change on streamflow in the Sierra Nevada, few have incorporated the effects of vegetation change [32].

In recent years, the California Sierra Nevada has been subject to an unprecedented drought [33] that has contributed to wide-scale lower montane forest die-off [34]. Portions of the Sierra Nevada have also been exposed to large wildfires with major stand-replacing patches, such as the 2013 Rim fire near Yosemite National Park [35]. As climate continues to warm, widespread vegetation type conversion within the Sierra Nevada is becoming an increasingly plausible scenario, yet the impact on streamflow generation remains largely unexplored. In this study, we investigated how forest-to-shrub type conversion in the lower montane forest zone of the California Sierra Nevada, in combination with projected climate change, may affect streamflow. We focused on the lower montane forest because the trailing (lower) edge of many forests is likely to be most susceptible to type conversion with climate warming and wildfire [36,37]. Simulation scenarios were modeled using a spatially distributed ecohydrologic model, Regional Hydro-Ecologic Simulation System (RHESSys), for a small and medium-sized watershed located at the Southern Sierra Critical Zone Observatory. As the future composition and distribution of shrubs species in the Sierra Nevada is unknown, multiple conversion scenarios were evaluated to test a range of potential effects on streamflow. Specifically, we examined the effect of type conversion to shrub species of three different sizes/leaf areas. In addition, we compared the effect of type conversion over the entire watershed to the effect of type conversion on only equatorial-facing aspects. Finally, each of the type conversion scenarios was simulated under a historical and a simple climate-warming scenario.

## Study Sites

The Sierra Nevada extends 400 km north to south in eastern California and has peak elevations ranging from less than 3000 m in the north to more than 4000 m in the south. Vegetation distributions in the Sierra Nevada are dominated by elevation gradients and transition from grasses and woodlands at the lowest elevations, to shrublands, lower montane forest, upper montane forest and alpine forest as elevations increase [38]. The Sierra Nevada is classified as having a Mediterranean climate, with warm dry summers and cool wet winters. As a consequence, the mountain range is characterized by low streamflow and low soil water availability during the summer when water demands are high for both human and ecological users [39]. Sierra Nevada provides water for over 23 million people and for agriculture in California’s Central Valley.

**Fig 1:**
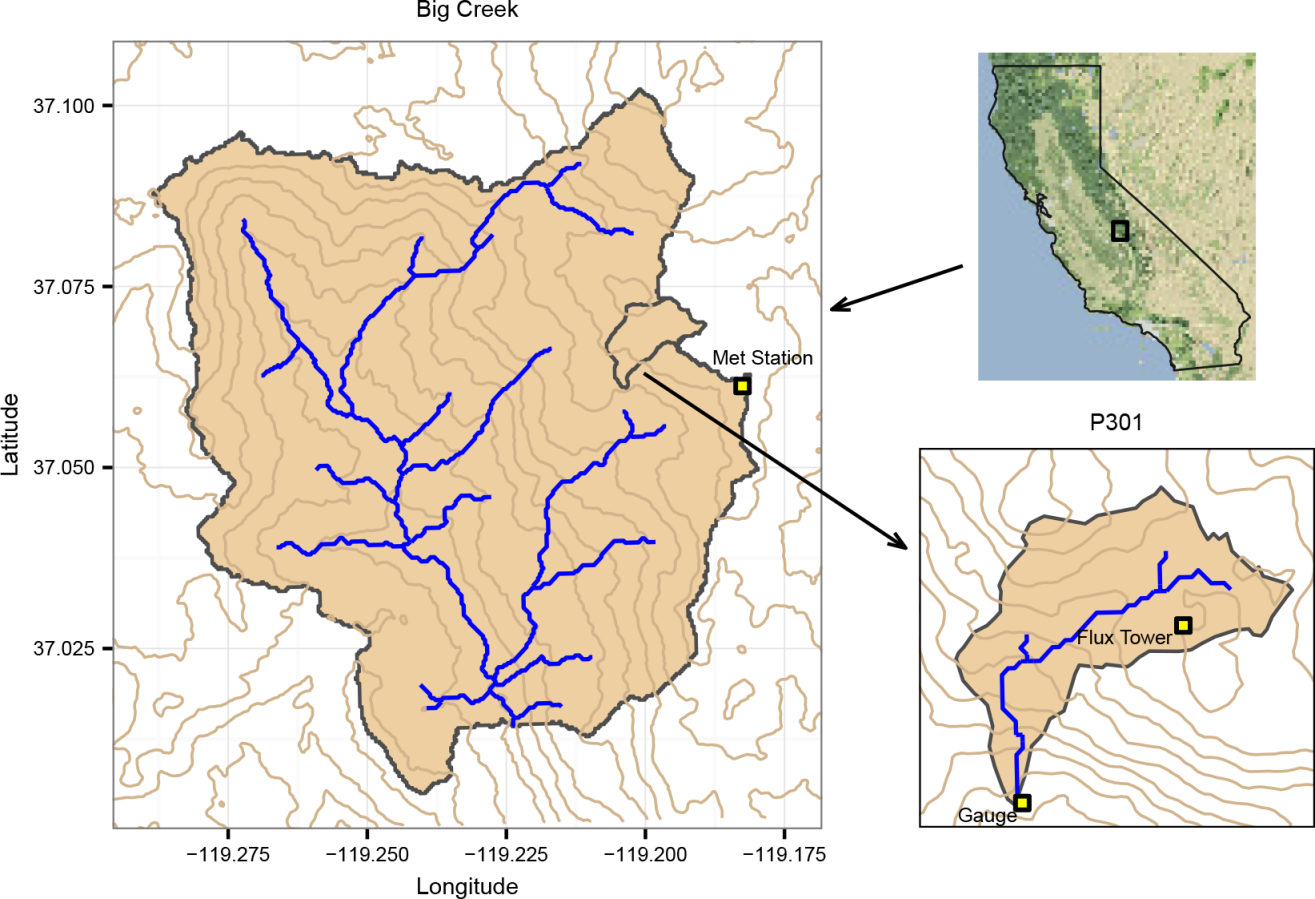
Location of P301 and Big Creek watersheds.

Vegetation type conversion was modeled in two watersheds, P301 and Big Creek, both located near Shaver Lake, California (Fig 1). P301 is a tributary of Providence Creek, which in turn is a tributary of Big Creek on its east flank. P301 is part of the highly instrumented Southern Sierra Critical Zone Observatory (CZO) and is 0.99 km^2^, with elevations ranging from 1790 m to 2115 m. Big Creek is a south-flowing river with an area of 65.7 km^2^ and encompassing a wider range of elevation, 957 m to 2344 m. The larger scale of the Big Creek watershed implies that it is likely to be less dominated by hillslope processes than P301, and potentially less sensitive to type conversion.

P301 is located at the rain-snow transition zone of the Sierra Nevada, with the snow fraction ranging from 35% to 60% of total precipitation [40]. Mean annual precipitation and mean annual streamflow for P301 are approximately 1320 mm and 528 mm, respectively [40]. The vegetation in P301 is composed primarily of Sierran mixed-conifer forest; which consists of white fir (*Abies concolor*), incense cedar (*Calocedrus decurren*s), ponderosa pine (*Pinus ponderosa*), Jeffery pine (*Pinus jeffreyi*), and sugar pine (*Pinus lambertiana*); as well as small proportions of meadows and chaparral shrublands (e.g. greenleaf manzanita (*Arctostaphylos patula*), mountain whitehorn (*Ceanothus cordulatus*)). The soils in P301 (Gerle-Cagwin) are granitic in origin [40] and generally have a very high water-holding capacity, estimated at ~3 m^3^/m^2^ by [41] when including regolith. These soils support the transpiration of deep-rooted vegetation year-round, with little vegetation shutdown during the cold winter season or the dry summer season [42]. The dominant aspect in P301 is equatorial-facing (74%).

Since the majority of Big Creek is located at lower elevations than P301, the watershed receives a lower proportion of snow compared to P301. Vegetation in Big Creek ranges from ponderosa pine forest with oaks at the lower elevations to Sierran mixed conifer forest at upper elevations. The two dominant soil types are Holland and Shaver. The lithology in Big Creek is classified as Mesozoic Plutonic and 67% of its slopes are equatorial facing.

Precipitation and temperature data for the calibration of P301 were obtained from a meteorological station located near the upper elevation of the watershed [40]. Discharge at the outlet of P301 was measured using two Parshall-Montana flumes, one each for high and low flows [40]. Evapotranspiration (ET) flux was measured via an eddy covariance tower located within the boundaries of P301 [42].

## Methodology

### RHESSys model

RHESSys is a spatially-distributed, daily time-step, ecohydrologic model that was developed to investigate the effects of land-cover and climate change on hydrologic and ecologic processes [43]. RHESSys has a hierarchical structure, with hydrologic and carbon cycling modeled at the patch scale (highest resolution), climate drivers organized at the zone level, and lateral routing of surface and subsurface water modeled at hillslope and watershed scales. Patches also include multiple vertical canopy layers. Incoming radiation is computed as a function of location, terrain and atmospheric variables (MtNClim) and radiation absorption and transmission is tracked through each canopy layer to the surface. The surface includes snowpack, litter and soil layers. The snow model is a quasi-energy budget model that accounts for the impact of canopy cover on snow accumulation, melt and sublimation. Precipitation is partitioned to snow and rain using air temperature. Evaporation and transpiration are modeled using Penman-Monteith [44]. Canopy interception is calculated as a function of vegetation size and vegetation type. Subsurface vertical moisture fluxes include infiltration and drainage through rooting and unsaturated zones. Lateral drainage of saturated water may be routed to the stream via surface flow, shallow subsurface flow, or groundwater. Flowpaths for the first two options are based on the topography of the watershed. Groundwater uses a parsimonious linear reservoir model to route water to the stream. The carbon cycling model in RHESSys includes estimates of photosynthesis, respiration and the allocation of net photosynthesis to leaves, stems and roots. RHESSys has been successfully implemented at a number of locations within the Sierra Nevada to investigate the impacts of climate variability and climate change [45–47]. Full details of the processes contained in RHESSys are provided in [43].

For this study we parameterized vegetation as standard classes; either conifer or shrubs rather than utilizing species-specific ecophysiological parameters. Parameters for a standard conifer or shrub were taken from RHESSys parameter libraries, with updates for maximum stomatal conductance and leaf water potential (*LWP*) as follows. Stomatal conductance (*gs*) in RHESSys was calculated based on a modified Jarvis multiplicative model [48]; where absorbed photosynthetically active radiation (*APAR*), carbon dioxide (*CO*_*2*_), *LWP*, vapor pressure deficit (*VPD*), and average and minimum temperature (*t*_*avg*_ and *t*_*min*_) are normalized multipliers used to scale maximum stomatal conductance (gs_max_);

$$gs=(m_{LWP}*m_{APAR}*m_{{CO}_{2}}*m_{VPD}*m_{t_{avg}}*m_{t_{min}})*{gs}_{max}.$$ (1)

To improve the parameterization of both trees and shrubs in the model, a field campaign was conducted during the spring and summer of 2014 in P301 to provide species-specific tree and shrub values for *gs*_*max*_ and the predawn *LWP* multiplier [49], as RHESSys simulations were expected to be sensitive to these values. White fir and incense cedar were sampled to represent conifers and greenleaf manzanita and mountain whitehorn were sampled for shrubs. Values of *gs*_*max*_ were derived from highest observed field measurements of stomatal conductance averaged over species samples on a given date. Higher values of *gs*_*max*_ were observed for shrubs (0.52 mol H_2_O m^-2^ s^-1^) than trees (0.12 mol H_2_O m^-2^ s^-1^), which is consistent with previous studies [50]. The values of *m*_*LWP*_ in Eq 1 were computed based on a power law relation developed between the observed normalized average *gs* (*gs*/*gs*_*max*_), which is equivalent to *m*_*LWP*_ when all other normalized multipliers are equal one, and observed predawn *LWP*, such that

$$m_{LWP}={\left(a*({LWP}_{predawn}-LWP_{thresh})+b\right)}^{p}$$ (2)

where *a* was 0.1, *b* was 1, *LWP*_*thresh*_ was -0.5 for trees and -0.3 for shrubs, and *p* was 9 for trees and 12 for shrubs (see Figure 27 in [49]). *m*_*LWP*_ has a minimum of 0 and a maximum of 1. The relation between *gs*_*max*_ and the other normalized multipliers in Eq 1 was held constant for both trees and shrubs.

Soils in P301 and Big Creek were initialized by allowing RHESSys to ‘spin up’ for 200 years. Spatial distributions of conifer carbon stores in P301 were based on 30 m estimates of leaf area index (LAI) generated from Light Detection and Ranging (LIDAR) [51] with allometric equations [52]. LIDAR LAI was not available for Big Creek. Instead, vegetation was initialized in Big Creek by growing conifers dynamically within RHESSys until mean carbon and nitrogen stores for the watershed approximately matched those from P301 (50 years). The spin-up approach for Big Creek allowed for spatial heterogeneity of LAI values at the patch level, however, the variability in LAI was much lower compared to the LIDAR approach (S1 Fig).

Soil depth in RHESSys represents the depth at which water is available to plant roots, and may include water-holding regolith that is not traditionally considered in soil classifications. Field based estimates of subsurface water availability for evapotranspiration in P301 suggest that roots can access water storage in excess of 3 m^3^/m^2^ [41], which would be consistent with soil depths in the model of approximately 5 m. For simulations in this study, the “hydrologically effective soil depth” was fixed at 5 m throughout the watershed, as there was an absence of data to guide soil depth distributions.

Subsurface drainage parameters in RHESSys are typically calibrated to account for uncertainty in subsurface drainage characteristics [53]. RHESSys was calibrated using a Monte Carlo approach to identify the optimal values of seven subsurface parameters. These parameters were evaluated against both observed streamflow and observed ET, with the top-10 calibrated parameter sets used for simulation scenarios. A full description of the calibration approach for P301 and Big Creek is provided in S1 Text.

### Scenarios

To generate a long-term record for simulations in P301 and Big Creek, we used a 55-year (1946–2000) adjusted daily temperature and precipitation record from a meteorological station near Grant Grove, which is located approximately 20 km south of P301 and at a similar elevation. Temperatures between Grant Grove and the local meteorological station in P301 were adjusted based on linear regression, with an R^2^ value of 0.89 and 0.85 obtained for the correlation of minimum and maximum daily temperatures, respectively. Precipitation for P301 and Big Creek was generated by scaling Grant Grove precipitation by 1.22, which reflected the change in mean annual precipitation between the two stations [47].

Three components were varied for each simulation scenario: amount of shrubland conversion in the watershed, LAI of the shrubs relative to trees, and a warming scenario. Baseline conditions for P301 and Big Creek were assumed to be 100% forest under historical (unchanged) temperatures. For each change scenario, we simulated the top ten parameter sets from calibration for 55 years under baseline conditions and compared modeled streamflow to simulations under modified conditions.

While future vegetation type conversion in the Sierra Nevada is likely to occur, specifics such as the location and timing of the conversion is more uncertain. There is also uncertainty regarding the specific species (both alien and native) that are likely to become established, which depending on species size, may have different effects on watershed processes. For this study, we considered two end member scenarios (100% shrub conversion and completely forested) and a plausible intermediate scenario, with shrub conversion only on equatorial-facing slopes.

Although we expect that conifers will have a higher LAI than shrubs, the precise difference between the two depends upon both site characteristics and the shrub and conifer species in question. To provide a general estimate of the consequences of shrub conversion, we considered a range of possible LAI reductions associated with shrub conversion; 1/2, 1/4 and 1/6 the LAI of each forest patch during calibration. These values provided a reasonable representation of the potential range of LAI differences between trees and shrubs [54]. Rooting depth was assumed to be constant between trees and shrubs, as sclerophyll shrubs are deeply rooted [55] and would likely have similar access to deep water as trees species.

Climate change scenarios were developed by adding a uniform 3°C warming to daily maximum and minimum temperatures [25]. Temperatures in the Western U.S. are predicted to increase between 1.1°C to 5°C by the end of the 21^st^ century [56], which is a reasonable timeframe for when widespread type conversion may be observed. A uniform 3°C increase in model temperatures falls directly in the middle of this projected increase. While climate projections indicate that summer temperatures in California may increase more than winter temperatures [57], we have selected a uniform increase to reduce the dimensionality of the simulations and focus on responses to an ‘average’ temperature change. In the western U.S and California, there is large uncertainty as to whether precipitation will change, with individual global climate models projecting both small increases and decreases [56,57]. Consequently, no changes in precipitation were assessed in this study.

## Results

The ensemble effect of simulating each of the top 10 parameter sets on mean annual streamflow in P301 and Big Creek; as well as associated hydrological variables mean annual evaporation, mean annual transpiration, and April 1 snow water equivalent (SWE); is shown in Table 1 for each simulation scenario. For clarity in presenting the results, we first discuss the effects of type conversion in the absence of climate change on streamflow, then the effects of climate change in the absence of type conversion on streamflow, and finally the combined effects of type conversion and climate change on streamflow. We then investigate the effect of type conversion on only equatorial-facing aspects of the watershed.

**Table 1:** Simulated mean annual streamflow, mean annual transpiration, mean annual evaporation, and April 1^st^ snow water equivalent (SWE) for vegetation and climate change scenarios.

Watershed	Temperature Change	Vegetation Type	Shrub LAI (Fraction of Tree LAI)	Mean Annual Streamflow (mm)	Mean Annual Transpiration (mm)	Mean Annual Evaporation (mm)	SWE- Apr 1 (mm)
P301	*0°C*	*Trees*	*NA*	*354*	*734*	*211*	*68*
	0°C	Shrub	1/2	353 (-1, 0%)	781 (47, 6%)	165 (-46, -22%)	73 (5, 7%)
	0°C	Shrub	1/4	420 (66, 19%)	739 (5, 1%)	139 (-72, -34%)	75 (7, 10%)
	0°C	Shrub	1/6	505 (151, 43%)	664 (-70, -10%)	125 (-86, -41%)	77 (9, 13%)
	3°C	*Trees*	NA	365 (11, 3%)	767 (33, 4%)	167 (-44, -21%)	4 (-64, -94%)
	3°C	Shrub	1/2	362 (8, 2%)	814 (80, 11%)	124 (-87, -41%)	4 (-64, -94%)
	3°C	Shrub	1/4	422 (68, 19%)	775 (41, 6%)	100 (-111, -53%)	4 (-64, -94%)
	3°C	Shrub	1/6	499 (145, 41%)	703 (-31, -4%)	91 (-120, -57%)	4 (-64, -94%)
Big Creek	*0°C*	*Trees*	*NA*	*445*	*667*	*184*	*26*
	0°C	Shrub	1/2	415 (-30, -7%)	736 (69, 10%)	146 (-38, -21%)	26 (0, 0%)
	0°C	Shrub	1/4	509 (64, 14%)	657 (-10, -1%)	126 (-58, -32%)	25 (-1, -4%)
	0°C	Shrub	1/6	600 (155, 35%)	569 (-98, -15%)	119 (-65, -35%)	24 (-2, -8%)
	3°C	*Trees*	NA	441 (-4, -1%)	690 (23, 3%)	165 (-19, -10%)	1 (-25, -96%)
	3°C	Shrub	1/2	413 (-32, -7%)	754 (87, 13%)	130 (-54, -29%)	1 (-25, -96%)
	3°C	Shrub	1/4	499 (54, 12%)	681 (14, 2%)	113 (-71, -39%)	1 (-25, -96%)
	3°C	Shrub	1/6	588 (143, 32%)	594 (-73, -11%)	107 (-77, -42%)	1 (-25, -96%)

Values in parentheses represent absolute change (mm) and percent change relative to the baseline scenario. Baseline scenario is indicated in italics. Mean annual precipitation over the simulation record was 1297 mm for both watersheds. Streamflow is defined as a depth (streamflow per year divided by watershed area).

### Effect of type conversion on watershed hydrology

For P301, simulation under baseline conditions of 100% forest cover and a historical temperature regime generated a mean annual streamflow yield of approximately 27% of annual precipitation, with mean annual transpiration and mean annual evaporation accounting for the remaining 57% and 16%, respectively (Table 1). Following 100% type conversion to shrublands, mean annual evaporation decreased relative to the baseline scenario as vegetation biomass and LAI decreased. This decrease, which ranged from 22% to 41%, was primarily associated with lower levels of canopy interception for the smaller shrubs. Type conversion both increased and decreased mean annual transpiration, depending on the size of the shrubs. For the shrub LAI 1/2 scenario, shrub transpiration increased 6% relative to the original trees. This increase in transpiration was partly due to a reduction in the evaporation of intercepted water from the vegetation canopy contributing to an increase in soil infiltration and subsequent water availability in the rooting zone of shrubs. It was also a result of stomatal conductance rates in shrubs being much higher than trees. The higher maximal conductance rates of shrubs more than compensated for reductions in transpiration due to shrubs having less leaf area. For the shrub LAI 1/4 scenario, higher conductance rates approximately offset lower leaf areas, producing similar transpiration rates as trees. For the shrub LAI 1/6 scenario, the reduction in leaf area was large enough to decrease mean annual transpiration levels by 10% compared to baseline conditions. Mean annual streamflow responses to type conversion ranged from no notable change for the shrub LAI 1/2 scenario to increases up to 151mm (43%) for the shrub LAI 1/6 scenario. For the former scenario, decreases in evaporation were balanced by increases in vegetation transpiration, while both evaporation and transpiration contributed to an increase in streamflow for the latter scenario.

**Fig 2:**
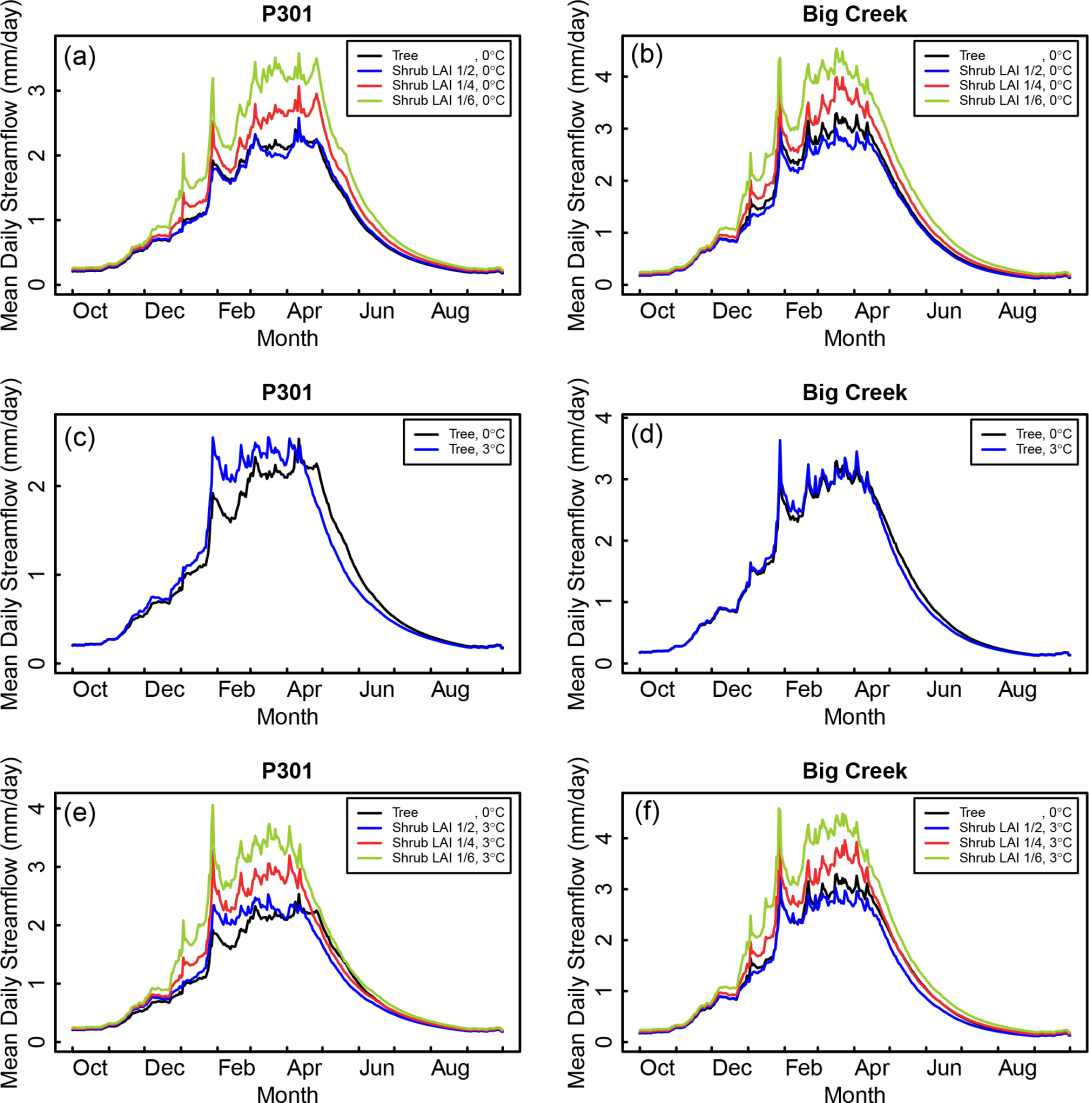
Mean daily streamflow for P301 and Big Creek. Comparison of mean daily streamflow under historical conditions (100% forest and historical temperatures) and the following change scenarios; (a) & (b) 100% type conversion to shrubs with LAI 1/2, LAI 1/4 and LAI 1/6; (c) & (d) 3°C temperature increase, and (e) & (f) both type conversion and temperature increase.

Fig 2A shows the simulated mean daily streamflow averaged by wateryear day (10 parameter sets times 55 years) for the P301 baseline scenario and the 100% type conversion scenarios. For the baseline scenario, mean daily streamflow increased throughout the early wet season, peaked in March and April, and then decreased sharply heading into summer. Following type conversion, the three LAI scenarios followed a similar inter-seasonal pattern as the baseline scenario, but the total amount of streamflow was scaled up.

**Fig 3:**
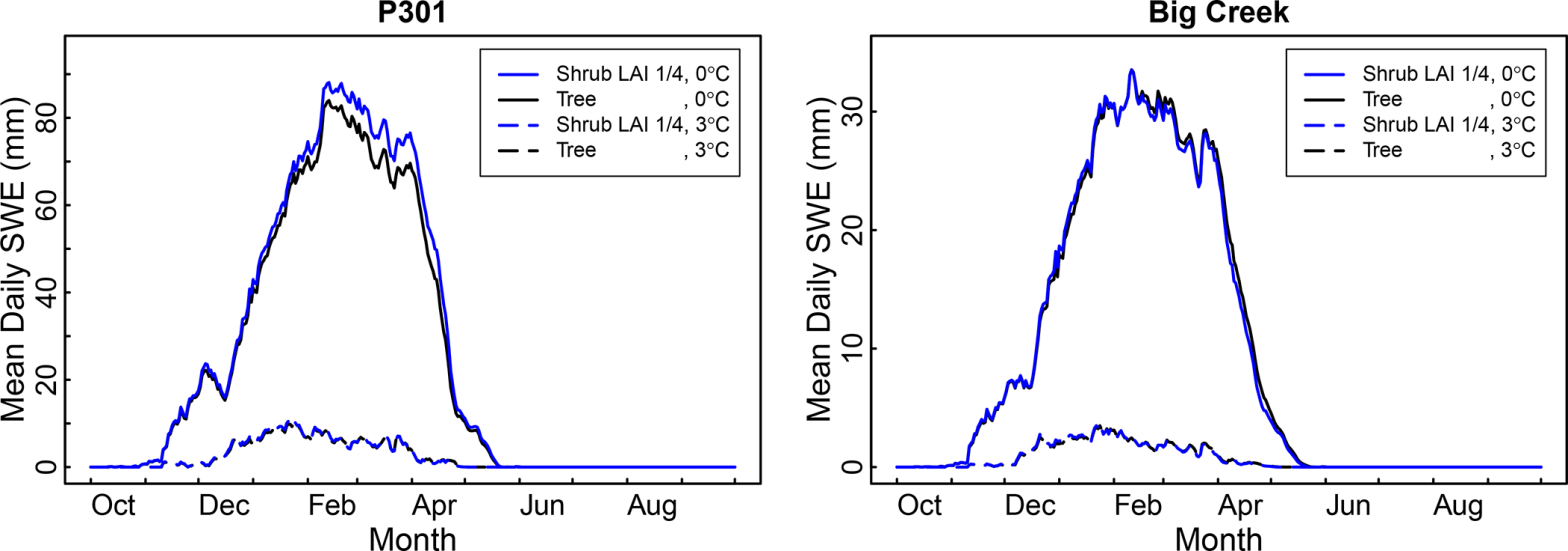
Mean daily snow water equivalent (SWE) for P301 and Big Creek. Four scenarios include baseline, 100% type conversion to shrubs with LAI 1/4, 3°C temperature increase, and both type conversion and temperature increase.

Type conversion to shrubs resulted in April 1 SWE levels increasing by up to 13% (Table 1). This increase in snowpack was produced by a decrease in the interception and subsequent sublimation of snowfall from the canopy. An example of the mean daily SWE for a LAI 1/4 scenario is shown in Fig 3.

Hydrologic partitioning of precipitation in Big Creek was similar to P301, but reflected some differences since the mean elevation of the watershed is lower than P301. Streamflow yield for the baseline scenario (34%) was higher in Big Creek compared to P301, whereas both transpiration and evaporation were lower (Table 1). Snowpack accumulation on April 1 was also lower in Big Creek (26 mm) than in P301 (68 mm). The smaller snowpack accumulation in Big Creek contributed to a higher proportion of early season flows in Big Creek compared to P301 (Fig 2A and 2B).

Following 100% type conversion from tree to shrubs in Big Creek, streamflow under the shrub LAI 1/2 scenario decreased 30mm (7%) compared to baseline conditions, in contrast to the negligible difference for P301 (Table 1). For the shrub LAI 1/6 scenario, streamflow in Big Creek increased 155mm (35%) compared to baseline, which is similar to P301 on an absolute basis.

### Effect of climate change on watershed hydrology

The most noticeable difference in the partitioning of precipitation in P301 following a 3°C increase in temperature was a nearly complete elimination of April 1 SWE (68 mm vs. 4 mm) (Table 1). The watershed shifted from being located in a rain-snow transition zone under historical temperatures to a rainfall-dominated zone under a 3°C warming scenario. A decrease in evaporation in P301 was produced by a reduction in snowpack sublimation, with the excess water contributing to a small increase in streamflow and transpiration (Table 1). More significant though, was a shift in the timing of streamflow (Fig 2C). Under the 3°C warming scenario, the center of mass for mean annual streamflow, which was defined as the day when cumulative mean daily streamflow equaled 50% of mean annual streamflow, advanced by 9 days, from March 25^th^ to March 16^th^. A corresponding decrease in streamflow at the end of the wet season was also observed (Fig 2C), which extended the length of the summer dry season.

Big Creek was less sensitive to climate warming than P301 since most of the watershed was located below the rain-snow transition zone. The total volume of streamflow was virtually unaltered under the 3°C scenario (Table 1) and the earlier shift in streamflow timing was less pronounced in Big Creek than in P301, with the center of mass for mean annual streamflow advancing 3 days, from March 16^th^ to March 13^th^ (Fig 2D).

### Effect of type conversion and climate change on watershed hydrology

The combined effect of tree-to-shrub type conversion and a 3°C temperature increase on mean annual streamflow was similar to the effects under the type conversion only scenario (Table 1). For larger shrub type conversion scenarios (LAI 1/2), mean annual streamflow showed little change, with a slight increase in post-conversion streamflow in P301 (2%) and moderate decrease in Big Creek (-7%). For smaller shrub type conversion scenarios (LAI 1/6), post-conversion mean annual streamflow showed large increases in mean annual streamflow in both P301 (41%) and Big Creek (32%).

The timing of streamflow under scenarios with both higher temperatures and type conversion was earlier compared to the baseline conditions (Fig 2E and 2F), as climate warming increased the proportion of streamflow that runs off during the winter instead of accumulating as snowpack. In the case of type conversion to the smallest shrubs (LAI 1/6), the magnitude of mean daily flows at the beginning of the wet season was approximately double the baseline streamflow. The start of the terminal recession curve at the end of the wet season also occurred earlier than under baseline conditions (Fig 2E and 2F). However, since type conversion to smaller shrubs generated higher flows than under baseline conditions, the timing of the terminal recession curves for a given streamflow magnitude was similar to baseline conditions, lessening the length of the summer drought period.

**Fig 4:**
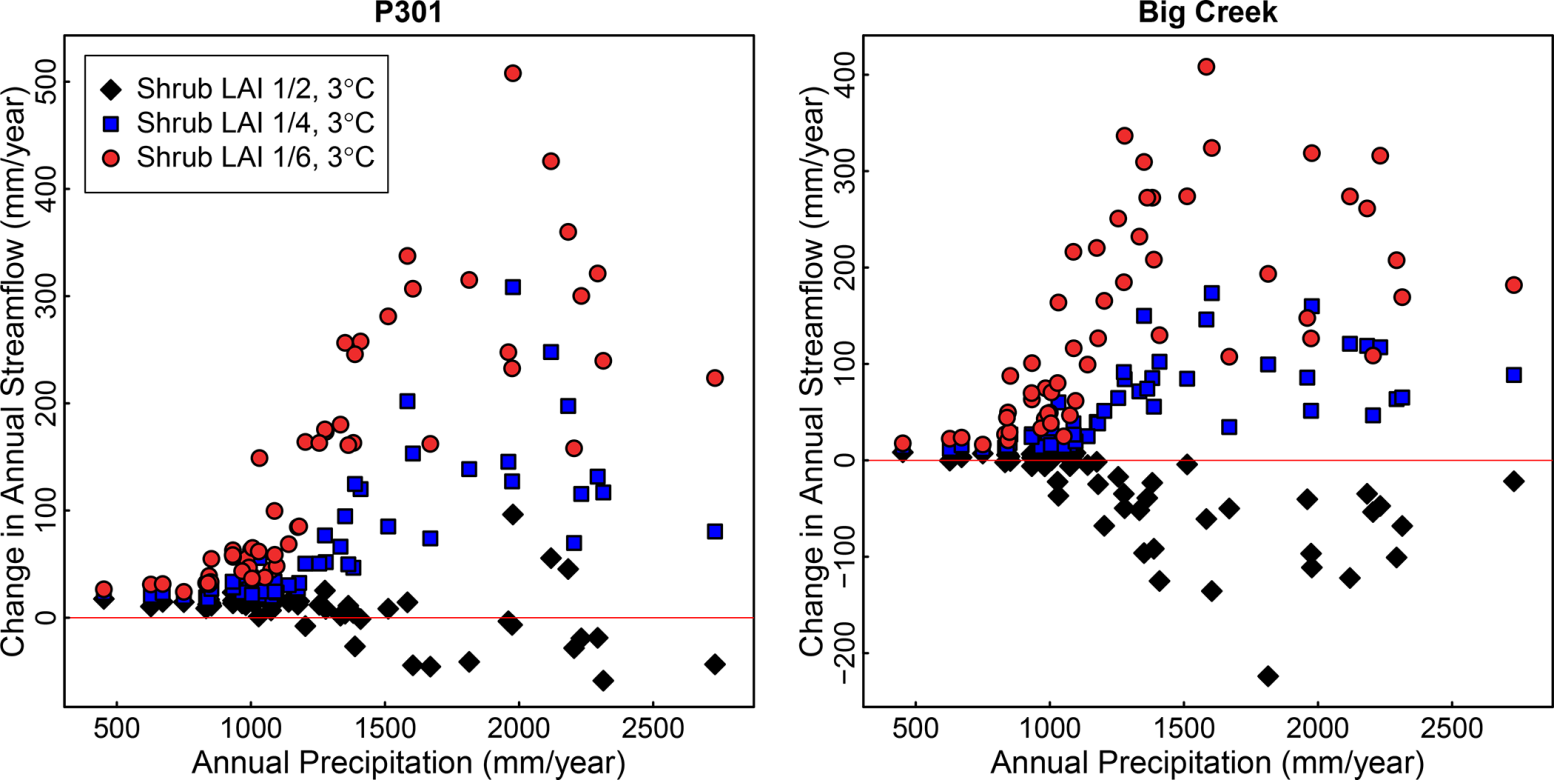
The difference between modeled annual streamflow under the baseline scenario and each post-conversion LAI scenario plotted against annual precipitation for P301 and Big Creek.

To understand how streamflow varies inter-annually, the difference between modeled annual streamflow under the baseline scenario and each of the post-conversion LAI scenarios was plotted against annual precipitation for P301 and Big Creek (Fig 4). The results show that post-conversion changes in streamflow varied considerably depending on wetness conditions. For all three shrub LAI scenarios, annual streamflow increased marginally under low (less than ~800 mm/year) precipitation conditions. During years when precipitation was above this threshold, annual streamflow both increased and decreased for the shrub LAI 1/2 scenario in P301 while only decreasing for the lower elevation Big Creek watershed. For both the shrub LAI 1/4 and shrub LAI 1/6 scenarios, annual streamflow increased following type conversion during high precipitation years.

### Effect of type-conversion aspect on watershed hydrology

We tested the effect of type conversion on equatorial-facing aspects to understand if streamflow response to type conversion may differ by aspect. To do this, we considered a scenario where type conversion occurred only on equatorial-facing aspects. Changes in streamflow for this equatorial-facing aspect scenario were compared to a scenario where type conversion occurred across the entire watershed (100% type conversion). To account for the difference in total area converted between the equatorial-facing aspect only scenario and the 100% type conversion scenario, we multiplied the change in streamflow for the 100% type conversion scenario by the proportion of watershed area with equatorial-facing aspects (73.8% for P301 and 67.4% for Big Creek). Thus, the 100% type conversion scenario can be considered an “aspect neutral” scenario that assumes streamflow change scales linearly with area converted and that streamflow generation is equivalent for equatorial and polar-facing aspects. Table 2 shows post-conversion changes of mean annual streamflow, mean annual evaporation and mean annual transpiration for the equatorial-facing aspect scenario and the aspect-neutral scenario.

**Table 2:** Change in hydrologic variables following type conversion for the equatorial-facing aspect scenario and the aspect-neutral scenario.

Watershed	Equatorial-Facing Aspects (%)	Shrub LAI (Fraction of Tree LAI)	Aspect Change Scenario	Change in Mean Annual Streamflow (mm)	Change in Mean Annual Transpiration (mm)	Change in Mean Annual Evaporation (mm)
P301	73.8	1/2	Aspect neutral	6	59	-64
			Equatorial-facing	9	60	-69
	73.8	1/4	Aspect neutral	50	30	-82
			Equatorial-facing	45	37	-82
	73.8	1/6	Aspect neutral	107	-23	-89
			Equatorial-facing	89	-5	-87
Big Creek	67.4	1/2	Aspect neutral	-22	59	-36
			Equatorial-facing	-22	64	-41
	67.4	1/4	Aspect neutral	36	9	-48
			Equatorial-facing	33	17	-53
	67.4	1/6	Aspect neutral	96	-49	-52
			Equatorial-facing	90	-39	-57

A 3°C increase was assumed for each scenario.

Overall, the effect of aspect on streamflow response was small (less than 13%) compared to the total post-conversion change in streamflow. In P301, type conversion to small shrubs (LAI 1/4 and 1/6) on equatorial-facing aspects with 3°C of warming increased mean annual streamflow less than the aspect-neutral type conversion scenario, while type conversion to larger shrubs (LAI 1/2) on equatorial-facing aspects increased mean annual streamflow slightly compared to the aspect-neutral type conversion scenario. The effect of type conversion aspect on streamflow change in Big Creek was similar, but less pronounced than in P301.

**Fig 5:**
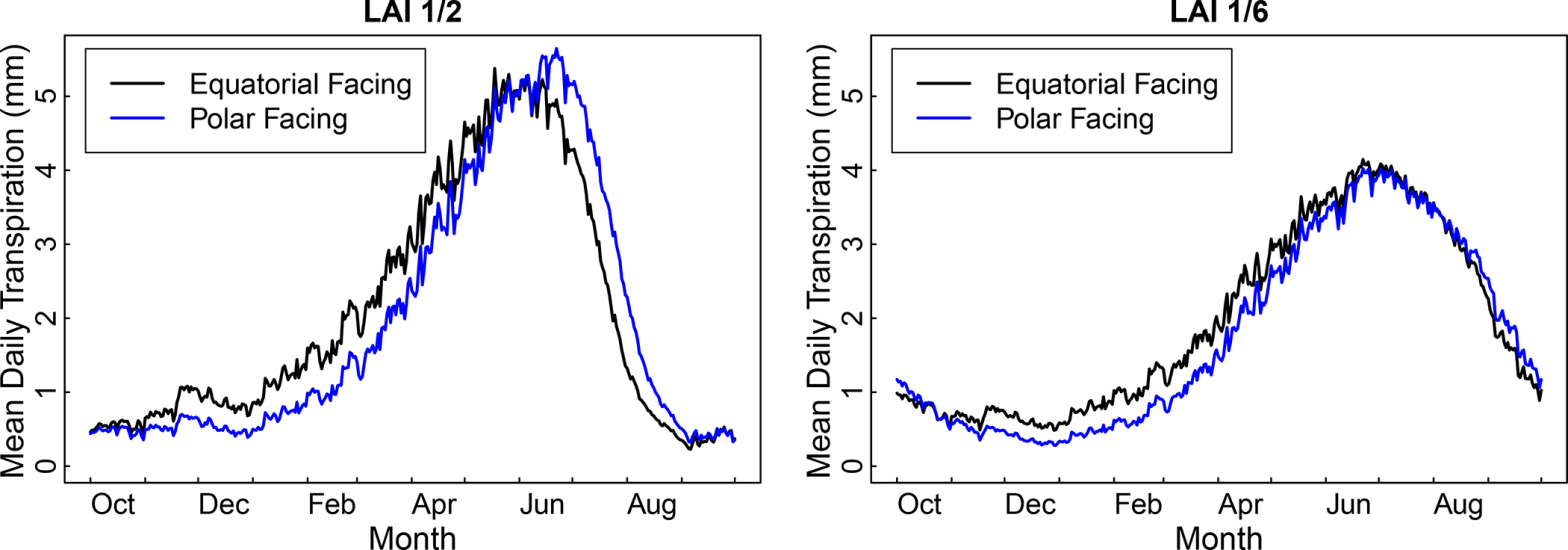
Mean daily transpiration for an equatorial- and polar-facing patch in P301. A 3°C increase was assumed for each scenario.

To understand why aspect amplifies streamflow change following type conversion to small shrubs but not following type conversion to large shrubs, we conducted two patch-level simulations of LAI 1/6 and two patch-level simulations of LAI 1/2. The simulations at each LAI level varied only by aspect, with one aspect being equatorial facing and the other aspect being polar facing. The patch had a slope of 16 degrees and a single patch was used in order to isolate aspect-related differences in hydrological behavior. The results showed that transpiration was higher for equatorial-facing aspects than for polar-facing aspects during the first part of the wateryear for both LAI scenarios (Fig 5). This was due to equatorial-facing aspects receiving more radiation than polar-facing aspects and water not being a limiting factor during the wet season. For the LAI 1/2 scenario, the relation between equatorial and polar-facing aspects reversed in early June, as the rooting zone storage in the equatorial-facing patch became water limited. The relative increase in transpiration for the polar-facing patch offset the higher transpiration of the equatorial-facing patch during the first part of the wateryear, producing similar levels of mean annual transpiration for both aspects (Table 2). For the LAI 1/6 scenario, overall transpiration levels were not as high as the LAI 1/2 scenario and the equatorial-facing patch only showed water limitation near the end of the wateryear. Consequently, mean annual transpiration for the equatorial-facing patch remained higher than for the polar-facing patch (Table 2).

## Discussion

Higher global temperatures and increased levels of disturbance are contributing to greater mortality in many forest ecosystems [10]. These same drivers can also limit forest regeneration, leading to vegetation type conversion [15,18,58]. For this study, we have examined the potential effects of tree-to-shrub type conversion in the lower montane forest of the Sierra Nevada on streamflow.

**Fig 6:**
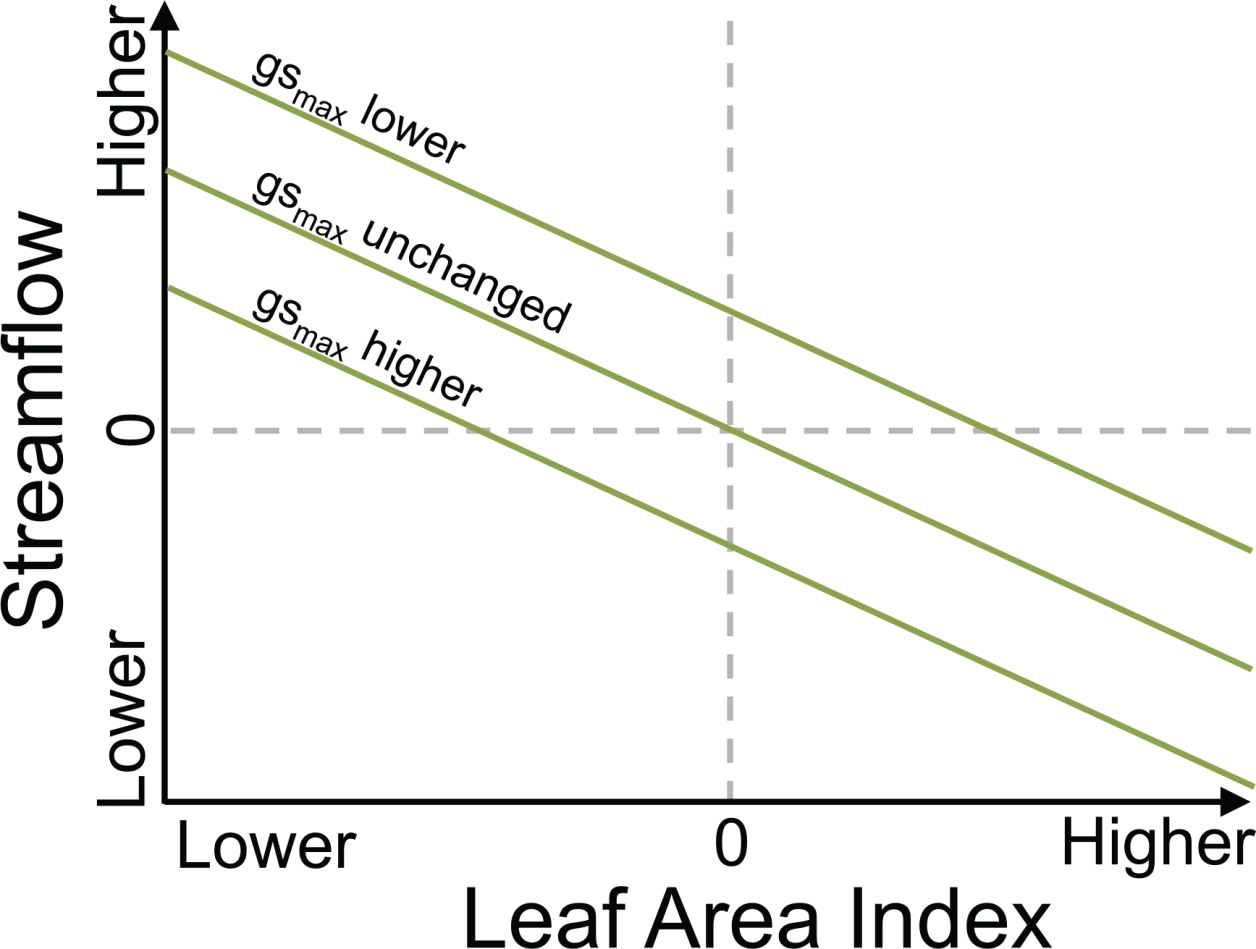
Conceptual model of streamflow as a function of LAI and stomatal conductance. Note the relation between LAI and streamflow is shown here as linear but other relations are possible. Type conversion from trees to shrubs tends to decrease LAI but also increase stomatal conductance, leading to variable effects on streamflow.

The modeling results demonstrated that while type conversion in lower montane forests may increase streamflow, the magnitude and direction of post-conversion streamflow change was variable and dependent on the LAI and stomatal conductance rates of the invading shrubs (Fig 6). High stomatal conductance rates for shrub species in the Sierra Nevada increased per-unit leaf area transpiration and helped to offset lower shrub LAIs. For example, for the shrub LAI 1/2 scenarios, changes in mean annual streamflow were negligible (P301) or negative (Big Creek) because increased shrub transpiration from higher stomatal conductance compensated for a corresponding decrease in shrub evaporation from lower LAIs (Table 1). This suggests that type conversion in lower montane forests, particularly below the rain-snow transition zone, could decrease Sierra Nevada water supplies if differences in LAI between pre- and post-conversion vegetation are small. On the other hand, increased mean annual streamflow was observed when shrub LAIs decreased below the compensating point for higher stomatal conductance, which was near shrub LAI 1/2 for P301 and between shrub LAI 1/2 and shrub LAI 1/4 for Big Creek. Following 100% tree-to-shrub type conversion below this compensating point, we found mean annual streamflow increased 12% to 19% for LAI 1/4 and 32% to 43% for LAI 1/6, depending on the watershed and temperature scenario.

Mean annual evaporation was positively correlated with LAI, such that a decrease in LAI also decreased mean annual evaporation. Mean annual transpiration was also positively correlated with LAI, however, only for a given level of stomatal conductance. Thus, similar to streamflow, estimates of transpiration change following type conversion will necessitate understanding how stomatal conductance varies between pre and post-conversion vegetation types.

A climate warming scenario of 3°C in the lower montane forest of the Sierra Nevada showed minimal effect on mean annual streamflow, but did generate an earlier shift in the timing of streamflow [6]. This shift was greatest in P301, which was initially centered in the temperature sensitive rain-snow transition zone. Higher flows were simulated during the early part of the wet season, as precipitation that previously accumulated as snowpack under historical temperatures instead ran off as streamflow under warmer conditions. This increase in early season streamflow may have significant impacts on flooding, stream geomorphology, and sediment production [29]. At the end of the wet season, streamflow declined earlier as snowmelt-driven baseflow was nearly eliminated. This reduction in soil water recharge from snowpack extends the length of the dry season and has the potential to impact vegetation productivity and water use [59].

Comparing the relative influence of both vegetation type conversion and climate warming on streamflow, model estimates suggested that vegetation change could have a greater impact on streamflow magnitude than the direct hydrologic impacts of increased temperatures. Temperature increases, however, may have a greater impact on streamflow timing.

Annual streamflow response to type conversion varied temporally with annual precipitation. During dry years when precipitation was below a threshold of 800 mm, post-conversion annual streamflow showed minimal increases for all shrub LAI scenarios because both large vegetation (trees) and small vegetation (shrubs) were able to adequately transpire all available precipitation [60]. This outcome reduces management options for exploiting type conversion as an approach for increasing water resources from the Sierra Nevada, as type conversion has little effect on annual streamflow during drought years when the need for water is greatest. Only when precipitation was above 800 mm and for the smaller shrub LAI scenarios did annual streamflow substantially increase compared to historical baseline conditions. During these wet years, there was sufficient water within the rooting zone for differences in the evaporational and transpirational capacity of trees and smaller shrubs to become significant [61]. This threshold response of streamflow change to annual precipitation is consistent with [22] who predicted streamflow change would be minimal in watersheds when annual precipitation was less than 500 mm.

Streamflow sensitivity to aspect for the two watersheds in this study ranged from small for scenarios where vegetation was not water-limited (e.g. LAI 1/6) to negligible for scenarios where vegetation was water-limited (e.g. LAI 1/2). This result occurred because annual transpiration was higher on equatorial-facing aspects than on polar-facing aspects in locations that were not water-limited, but similar for equatorial-facing and polar-facing aspects in locations that were water-limited. These results suggest that type conversion aspect may be important to account for in less water-limited environments, such as those with smaller vegetation, deeper soils or less evaporative demands.

A key assumption in this study was that the deep soil depths observed in P301 were uniformly distributed throughout the entire P301 and Big Creek watersheds. However, it should be noted that if soils depths were shallower, streamflow change following type conversion would likely differ as less water storage capacity in the soils would cause transpiration to be water-limited more frequently [49]. It was further assumed that shrubs have a similar rooting depth to trees, which may not be true for some species (e.g., [62]). In cases where shrub rooting depths are substantially shallower than trees, changes in mean annual streamflow may be different than observed in this study, as the amount of available water storage in the rooting zone is reduced. A similar result may occur under type conversion scenarios to grasses. While not explicitly considered in this study, under certain climate and disturbance regimes, type conversion to grasses may provide a more stable vegetation state than shrubs [20,58]. The effects of vegetation transformation to grasses in lower montane forests is beyond the scope of this paper, but may have a considerable impact on future Sierra Nevada streamflow. We also assumed that our estimates of stomatal conductance parameters derived from field measurements at the study sites were generalizable. Further assessment and ongoing monitoring under a wider range of sites and conditions would reduce this uncertainty.

While vegetation in the lower montane forest is expected to be sensitive to fire-driven type conversion [36], streamflow generated from the lower montane forest only constitutes a fraction of the total streamflow originating in the Sierra. The largest percentage of runoff in the Sierra Nevada is produced at elevations above the lower montane forest (e.g. upper montane forest, subalpine forest, tundra) [63], where snowpacks are larger and ET rates are lower. A comprehensive assessment of the effects of type conversion on Sierra Nevada water supplies will necessitate understanding how vegetation may shift across the entire elevation gradient of the Sierra Nevada.

In conclusion, this study has examined the effect of tree-to-shrub type conversion in the lower montane forest of the Sierra Nevada on streamflow. Model results indicated that streamflow may show negligible change or small decreases following type conversion when the difference between tree and shrub leaf areas is small, partly due to the high stomatal conductivity and the deep rooting depth of shrubs. In contrast, streamflow may increase when post-conversion shrubs have a small leaf area relative to trees. Vegetation type conversion was shown to have a greater effect on streamflow magnitude than climate warming, however, climate warming had a greater effect on streamflow timing. Tree-to-shrub type conversion increased streamflow only marginally during dry years, with most streamflow change observed during wetter years. These modeling results underscore the importance of accounting for changes in vegetation communities, including future LAI values under altered climates, to accurately characterize future hydrologic regimes for the Sierra Nevada.

## Supporting Information

S1 Fig Density plot of patch-level LAI values for P301 and Big Creek watersheds. (EPS)

S1 Text Description of model calibration. (PDF)
