# Supplementary material for: Effect of Tree-to-Shrub Type Conversion in Lower Montane Forests of the Sierra Nevada (USA) on Streamflow
Source: PLoS One. 2016 Aug 30;11(8):e0161805. doi: 10.1371/journal.pone.0161805 (PMC5004902; doi:10.1371/journal.pone.0161805)
Supplement: S1 Text — (PDF) [file pone.0161805.s002.pdf]

S1 Text: Model Calibration

A Monte Carlo approach using uniform probability distributions was used to calibrate seven sub-surface drainage parameters. The range for each of these parameters is provided in Table A. RHESSys was calibrated against the observed streamflow record in P301 and against the observed ET from the eddy covariance tower. This additional calibration measure was used to falsify parameter sets [1] and assure that the model was getting a good fit for right reasons [2]. Seven years of streamflow data (wateryears 2004-2010) and four years of ET data (wateryears 2009-2012) were used for calibration. For both calibrations, 4000 identical parameter sets were sampled.

Table A. Calibration range of model parameters using a uniform probability distribution.

Parameter	Units	Calibration Range
Saturated hydraulic conductivity (K)	m day^{-1}	1 - 150
Decay rate of hydraulic conductivity with depth (m)	m^{-1}	0.8 - 6
Soil air entry pressure (pa)	m	1 - 10
Pore size index (po)	-	0.3 - 1.2
Percent of precipitation that goes to groundwater (GW1)	%	0.0001 - 0.4
Percentage of groundwater that moves to the stream (GW2)	%	0.0001 - 1
Rooting Depth (rd)	m	3 - 5

Ten parameter sets were selected for simulation based on the parameter sets that had the best-calibrated fit for streamflow and were also behavioral for ET. Modeled streamflow was compared to observed streamflow using a composite objective function, Accuracy,

$$Accuracy = NSE * NSE_{log} * (1 - |PerErr|), \quad (S1)$$

which is the product of the objective functions Nash Sutcliffe Efficiency (NSE), NSE_{log}, and total percent error (PerErr). NSE is a measure of model fit for peak flows, NSE_{log} is a measure of model fit for medium/low flows and PerErr is a measure of the difference in total modeled streamflow volumes. Values of accuracy range from 0 to 1, with 1 indicating perfect fit and 0 indicating poor fit. This composite objective function is similar to the objective function recommended by Moriasi et al. [3], but with NSE_{log} substituted for the RMSE-observations standard deviation ratio (RSR). NSE_{log} was substituted because it is more sensitive to low flows and low flows constitute a substantial proportion of streamflow in MTEs. The individual objective functions are defined as:

$$NSE = 1 - \frac{\sum(Q_{obs} - Q_{mod})^2}{\sum(Q_{obs} - \overline{Q_{obs}})^2} \quad (S2)$$

$$NSE_{log} = 1 - \frac{\sum(\log(Q_{obs}) - \log(Q_{mod}))^2}{\sum(\log(Q_{obs}) - \log(\overline{Q_{obs}}))^2} \quad (S3)$$

$$PerErr = \frac{(\overline{Q_{mod}} - \overline{Q_{obs}})}{\overline{Q_{obs}}} \quad (S4)$$

where Q_{obs} is observed streamflow and Q_{mod} is modeled streamflow. Values of NSE and NSE_{log} range from negative infinity to 1, however, only parameter sets with values above 0 were considered behavioral and included further in the study. Similarly, parameter sets with PerErr values less than -1 or greater than 1 were considered non-behavioral and excluded from the analysis. Modeled ET was compared to observed ET using NSE, PerErr, and Pearson's Correlation coefficient (r), where r values greater than 0.5 were considered behavioral.

Model calibration results for the top 10 parameter sets are provided in Table B and Fig A. Overall, the model did a better job of replicating streamflow than ET, with the top

parameter sets having Accuracy, NSE and NSE_{log} streamflow values up to 0.41, 0.59 and 0.77, respectively (Table 1). Modeled PerErr for annual streamflow totals during calibration was generally very low, ranging from -0.14 to 0.06. The model captured the seasonality of streamflow; matching winter peaks, recession and summer low flow periods (Fig A). In some wateryears (e.g. 2008-2010), there was a mismatch in the timing of late season flows, with the model generating earlier runoff than observed due to an underestimation of the partitioning of incoming precipitation between snow and rain. RHESSys uses air temperature to partition precipitation and errors with this approach may be large when air temperatures are near zero degrees [4]. While misclassification of incoming precipitation in a particular year will lead to poor streamflow and evapotranspiration estimates for that year, the impact and bias on simulations of long-term ecohydrologic fluxes is likely small. In general, model performance was consistent with the performance of other hydrologic modeling studies in the Sierra Nevada [5,6]. The model also did a reasonable job of capturing the seasonal pattern and magnitude of ET, with the exception of wateryear 2011 where the model overpredicts spring ET and underpredicts summer ET. Again this error may be due to misclassification of incoming precipitation.

Table B. Calibration results for the top 10 parameter sets in P301.

Variable	Objective Function	Value
Streamflow	NSE daily	0.48 to 0.59
	NSE_{log} daily	0.67 to 0.77
	PerErr (annual)	-0.14 to 0.06
	Accuracy	0.35 to 0.41
ET	NSE daily	0.46 to 0.52
	Correlation Coeff (r)	0.54 to 0.58
	PerErr (annual)	-0.04 to 0.00

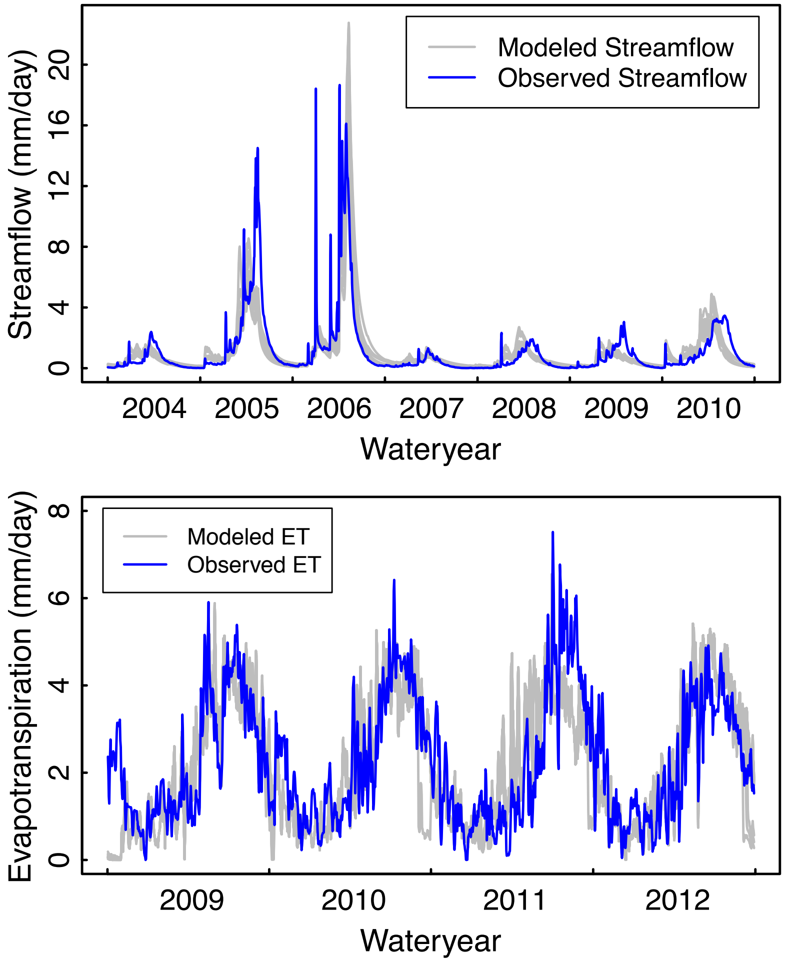

Fig A. Modeled streamflow vs. observed streamflow and modeled patch-level ET vs. observed flux tower ET in P301. Blue line represents the observed variable and gray lines represent modeled output for the ten top parameter sets.

Big Creek is an ungauged stream and no streamflow data were available to directly calibrate the RHESSys model. Instead, parameter sets from the top calibrations in P301 were transferred to Big Creek. The geological classification of Big Creek was the same as P301, Mesozoic Plutonic Rocks, and we assumed that subsurface drainage parameters were likely to be similar, following Tague et al. [7].

References

1. Son K, Sivapalan M. Improving model structure and reducing parameter uncertainty in conceptual water balance models through the use of auxiliary data. Water Resour Res. 2007;43: W01415. doi:10.1029/2006WR005032

2. Kirchner JW. Getting the right answers for the right reasons: Linking measurements, analyses, and models to advance the science of hydrology. Water Resour Res. 2006;42: W03S04. doi:10.1029/2005WR004362

3. Moriasi DN, Arnold JG, Van Liew MW, Bingner RL, Harmel RD, Veith TL. Model evaluation guidelines for systematic quantification of accuracy in watershed simulations. Trans ASABE. 2007;50: 885–900. doi:10.13031/2013.23153

4. Lundquist JD, Neiman PJ, Martner B, White AB, Gottas DJ, Ralph FM. Rain versus snow in the Sierra Nevada, California: Comparing doppler profiling radar and surface observations of melting level. J Hydrometeorol. 2008;9: 194–211. doi:10.1175/2007JHM853.1

5. Dettinger MD, Cayan DR, Meyer MK, Jeton AE. Simulated hydrologic responses to climate variations and change in the Merced, Carson, and American River basins, Sierra Nevada, California, 1900–2099. Clim Change. 2004;62: 283–317. doi:10.1023/B:CLIM.0000013683.13346.4f

6. Null SE, Viers JH, Mount JF. Hydrologic Response and Watershed Sensitivity to Climate Warming in California's Sierra Nevada. PLOS ONE. 2010;5: e9932. doi:10.1371/journal.pone.0009932

7. Tague CL, Choate JS, Grant G. Parameterizing sub-surface drainage with geology to improve modeling streamflow responses to climate in data limited environments. Hydrol Earth Syst Sci. 2013;17: 341–354. doi:10.5194/hess-17-341-2013
